# Three-dimensional finite element analysis for internal fixation of mandibular condylar fractures: a scoping review

**DOI:** 10.1007/s00068-026-03259-5

**Published:** 2026-07-08

**Authors:** Yasumasa Kakei, Robert Sader, Philipp Ebener, Atsuki Tanaka, Masaya Akashi, Ryosuke Kuroda, Philipp Thoenissen

**Affiliations:** 1 Department of Oral and Maxillofacial Surgery, Kobe University Graduate School of Medicine, 7-5-2 Kusunoki-cho, Chuo-ku, Kobe, 650-0017 Japan; 2 Department of Oral, Cranio-Maxillofacial and Plastic Facial Surgery, Goethe University Frankfurt, University Hospital, Theodor-Stern-Kai 7, 60590 Frankfurt am Main, Germany; 3 Department of Orthopaedic Surgery, Kobe University Graduate School of Medicine, Kobe, Japan

**Keywords:** Finite element analysis, Mandibular condylar fracture, Internal fixation, Scoping review, Biomechanics, PRISMA-ScR

## Abstract

**Purpose:**

Three-dimensional finite element analysis (3D-FEA) has emerged as an essential computational tool for evaluating the biomechanical behaviors of internal fixation systems for mandibular condylar fractures. This scoping review maps and synthesizes the existing literature on 3D-FEA studies investigating the internal fixation of mandibular condylar fractures and explores, in a descriptive manner, differences in methodological focus between European and non-European research centers.

**Methods:**

PubMed and Cochrane Library databases were systematically searched following PRISMA-ScR guidelines. Original English-language research articles using 3D-FEA to evaluate internal fixation of mandibular condylar fractures were included. Data were extracted using a standardized charting form.

**Results:**

Thirty-six studies met the inclusion criteria. European studies (*n* = 12) predominantly focused on development of novel plate designs and mechanical testing protocols, whereas Asian and other nationality studies (*n* = 24) emphasized material comparisons between titanium and bioabsorbable systems, patient-specific modeling, and loading condition analysis. Consensus findings include: (1) double-plate fixation provides superior stability; (2) among 3D plate designs, trapezoid plates demonstrate the best performance for subcondylar and condylar base fractures, whereas alpha and lambda plates may be preferable for condylar neck fractures; (3) plate positioning along the posterolateral ramus border and anterolateral sigmoid notch (A+B configuration) offers optimal biomechanical performance; and (4) contralateral molar clenching produces the highest stress concentrations.

**Conclusion:**

Across the included FEA studies, the A+B double-plate configuration was the most consistently reported biomechanically favorable approach for both titanium and bioabsorbable systems; the trapezoid plate performed best for subcondylar and condylar base fractures, and two-screw osteosynthesis was effective for condylar head fractures. As these findings derive from computational models, clinical validation remains necessary. Future research should focus on patient-specific modeling, clinical outcome validation, and standardization of methodological protocols.

**Supplementary Information:**

The online version contains supplementary material available at 10.1007/s00068-026-03259-5.

## Introduction

Mandibular condylar fractures represent 25%-35% of all mandibular fractures and remain one of the most challenging injuries in maxillofacial traumatology [1, 2]. The anatomical complexity of the temporomandibular joint region, combined with the functional demands of mastication, necessitate precise surgical planning and optimal fixation strategies. The classification system proposed by Loukota et al. [3] categorizes condylar process fractures into three types: condylar head fractures, condylar neck fractures, and condylar base fractures. This classification has been widely adopted to standardize fracture modeling and facilitate inter-study comparisons.

Finite element analysis (FEA) is a computational technique that divides complex geometric structures into smaller simpler elements connected at nodes, thereby allowing numerical solution of partial differential equations governing mechanical behavior [4]. In maxillofacial biomechanics, FEA enables non-invasive evaluation of stress and strain distributions that would not be possible to measure directly in vivo. Advances in imaging technology and computational power have resulted in the methods becoming increasingly sophisticated, evolving from simplified two-dimensional models to highly detailed three-dimensional reconstructions incorporating patient-specific anatomy and heterogeneous material properties [5].

Open reduction and internal fixation (ORIF) using various plate configurations has demonstrated superior functional outcomes compared with closed treatment for displaced condylar neck and base fractures [6, 7]. However, the optimal plate design, positioning, and material selection remain subjects of ongoing investigation. Over the past two decades, three-dimensional FEA (3D-FEA) has emerged as a powerful computational method for evaluating stress distribution, strain patterns, and displacement characteristics in fixated fracture models [6, 8].

The clinical significance of FEA in condylar fracture management lies in its ability to predict the mechanical performance of different fixation strategies before clinical implementation. This predictive capability is particularly valuable given the anatomical constraints of the condylar region, where surgical access is limited and complications such as facial nerve injury, malocclusion, and temporomandibular joint dysfunction remain important concerns [9]. By identifying optimal plate configurations and positioning strategies through computational analysis, surgeons can make more informed decisions tailored to individual fracture patterns.

A preliminary search of PubMed and the Cochrane Database revealed no existing scoping reviews or systematic reviews specifically addressing the use of FEA in mandibular condylar fracture fixation. While individual studies have contributed valuable insights, the heterogeneity in methodological approaches, outcome measures, and reporting standards makes it challenging to synthesize findings and identify knowledge gaps.

The objective of this scoping review is therefore to map and synthesize the existing literature on studies using 3D-FEA to investigate the internal fixation of mandibular condylar fractures. The specific aims include: (1) identifying the types and extent of available evidence; (2) summarizing key research themes, methodological approaches, and findings; (3) exploring, in a descriptive and exploratory manner, potential differences in research focus between studies conducted at European institutions and those conducted elsewhere; and (4) identifying gaps in the existing literature to inform future research priorities.

## Methods

This scoping review was conducted in accordance with the Joanna Briggs Institute (JBI) methodology for scoping reviews [10] and is reported following the Preferred Reporting Items for Systematic Reviews and Meta-Analyses extension for Scoping Reviews (PRISMA-ScR) guidelines [11].

### Protocol and registration

The review protocol was developed a priori, and because of the nature of this scoping review, formal registration was not undertaken.

### Eligibility criteria

The eligibility criteria were established using the Population, Concept, and Context (PCC) framework:

**Population:** Computational models of mandibular condylar fractures (head, neck, or base fractures).

**Concept:** Three-dimensional FEA evaluating internal fixation (plates, screws, or a combination).

**Context:** Biomechanical evaluation of fixation stability, stress distribution, strain patterns, or displacement,

**Inclusion Criteria** Original research articles published in English using 3D-FEA methodology to evaluate internal fixation of mandibular condylar fractures and reporting biomechanical outcomes (stress, strain, displacement) were included.

**Exclusion Criteria** Review articles, editorials, letters, and conference abstracts; studies using only 2D FEA; studies focusing solely on temporomandibular joint prostheses without fracture fixation; studies evaluating mandibular fractures other than the condylar region; and articles not available in English were excluded.

### Information sources and search strategy

A comprehensive search of PubMed and the Cochrane Library was conducted from database inception to 31 December 2025; the final search was executed on 3 March 2026. The search strategy was developed in consultation with a medical librarian, and combined Medical Subject Heading (MeSH) terms with free-text keywords. The PubMed search strategy was: (“Finite Element Analysis”[MeSH] OR “Finite Element Analysis”[tiab] OR “finite element method”[tiab] OR “finite element model”[tiab] OR “finite element study”[tiab] OR “finite element evaluation”[tiab] OR “finite element”[tiab] OR FEA[tiab] OR FEM[tiab]) AND (“Mandibular Condyle”[MeSH] OR “Mandibular Condyle”[tiab] OR “condylar process”[tiab] OR “condylar neck”[tiab] OR “condylar base”[tiab] OR “condylar head”[tiab] OR “subcondylar”[tiab] OR “condyle neck”[tiab] OR “condyle fracture”[tiab] OR “condylar fracture”[tiab]) AND (“Fracture Fixation, Internal”[MeSH] OR “Bone Plates”[MeSH] OR “internal fixation”[tiab] OR osteosynthesis[tiab] OR “plate fixation”[tiab] OR “screw fixation”[tiab] OR miniplate*[tiab] OR “rigid fixation”[tiab] OR “plate osteosynthesis”[tiab] OR ORIF[tiab]). Additionally, the reference lists of all included studies and relevant review articles were hand-searched to identify any other eligible studies.

### Study selection

**Fig. 1:**
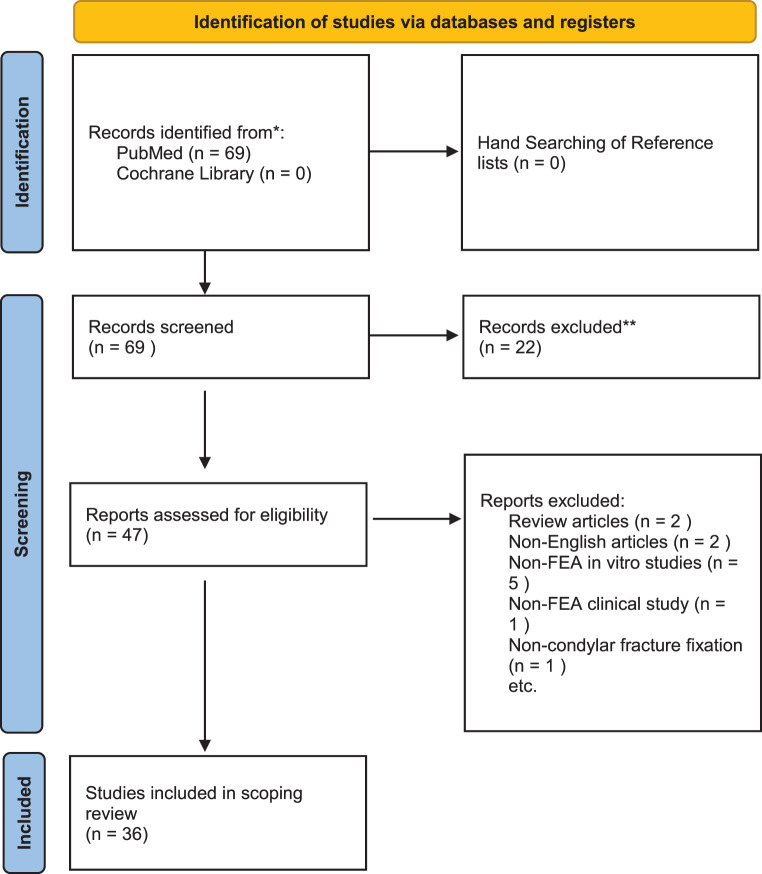
PRISMA 2020 flow diagram for scoping reviews showing the study selection process

All retrieved citations were imported into EndNote reference management software and duplicates were removed. Two reviewers independently screened the titles and abstracts for potential eligibility. Full-text articles of potentially relevant studies were retrieved and independently assessed against the inclusion and exclusion criteria by both reviewers. Any disagreements were resolved through discussion or consultation with a third reviewer. The study selection process is presented in a PRISMA flow diagram (Fig. 1).

### Data charting

Data were extracted independently by two reviewers using a standardized charting form. The following information was extracted: study characteristics (authors, year, country); FEA methodology (software, mesh characteristics, material properties); fracture characteristics; fixation systems evaluated; loading conditions; outcome measures; and key findings. The standardized data-charting form used for data extraction is provided as Supplementary Material (Supplementary Table S1).

### Synthesis of the results

Owing to heterogeneity in the study designs, methodological approaches, and outcome measures, a narrative synthesis approach was employed. Studies were grouped and analyzed according to geographical region, research focus, and key findings. For the geographic comparison, studies were categorized as European or non-European (other regions) on the basis of the country of the institution at which the study was performed, as indicated by the authors’ affiliations. This grouping was used solely for descriptive, exploratory mapping of differences in research focus and was not intended as a formal statistical comparison.

## Results

### Study selection

The database search identified 69 records from PubMed and no records from the Cochrane Library. The 69 records were screened by title and abstract, resulting in 22 being excluded. The PRISMA flow diagram illustrating the study selection process is presented in Fig. 1. The full texts of the remaining 47 articles were assessed for eligibility and 11 were excluded (two review articles, two non-English articles, five non-FEA in vitro studies, one non-FEA clinical study, one study primarily evaluating fixation of non-condylar fracture). Hand-searching of the reference lists did not identify any additional studies. Therefore, a total of 36 studies met the inclusion criteria and were included in this scoping review.

### Characteristics of the included studies

The 36 included studies were published between 2002 and 2025. Twelve originated from European institutions (Austria, Poland, Switzerland, Italy, Germany, and Ukraine) and 24 originated from Asian, South American, Middle Eastern, or African institutions (Japan, China, Taiwan, Korea, India, Syria, Brazil, Turkey, and Egypt). The characteristics of all the included studies are summarized in Table 1.

**Table 1:** Characteristics of the included studies

Ref	Author, Year	Country	Fracture Type	Plate Types	Materials	Key Findings
[1]	Aquilina, 2013	Australia	Condylar base	Single, double parallel, double offset	Titanium	Two parallel miniplates superior to single plate; parallel configuration superior to offset
[6]	Li, 2022	China	Condylar base	Position A, B, C	Ti, u-HA/PLLA	A+B configuration optimal for both Ti and bioabsorbable
[8]	Wagner, 2002	Austria	Condylar neck	Various configurations	Titanium	Foundational FEA methodology; heterogeneous material properties important
[12]	Albogha, 2018	Japan	Condylar base	5 designs: lambda, strut, delta, rhombic, trapezoid	Titanium	Trapezoid best; delta high screw loosening risk; patient-specific FEA
[13]	Gupta, 2023	India	Condylar base	Single, double, trapezoid, lambda, strut	Titanium	Orthotropic bone properties; 6 load cases analyzed
[14]	Darwich, 2016	Syria	Condylar base	5 techniques: single, double, trapezoid, delta, lambda	Titanium	Trapezoidal plate recommended; optimal stability with less bone surface
[15]	Hijazi, 2016	Syria	Condylar base	Various	Titanium	LMOL produces highest stress; loading condition analysis
[16]	Huang, 2021	Taiwan	Condylar base	Miniplates various positions/lengths	Titanium	Two plates superior; longer plates better than shorter; posterior placement reduces sliding distance compared to anterolateral
[17]	Kozakiewicz, 2014	Poland	Condylar neck	A-shape plate vs. 9-hole trapezoid plate	Titanium	A-shape plate provides greater stability than trapezoid plate (64.5 vs 116.3 μm displacement); multipoint fixation and reinforced bars along compression/traction lines; applicable to all levels of neck fractures
[18]	Liokatis, 2022	Germany	Condylar neck and base	Lambda plate	Titanium	Lambda plate adequate for neck fractures with cranial placement; inadequate for base fractures; second plate at sigmoid notch recommended for base fractures
[19]	Schönegg, 2024	Switzerland	Condylar head	Magnesium screws (2 vs 3)	Magnesium	Three magnesium screws recommended; 30–46% von Mises stress reduction vs two screws; two-screw configuration stress exceeded tensile strength
[20]	Parascandolo, 2010	Italy	Condylar base	Two load-sharing plates	Titanium	Two-plate fixation along compressive (posterior border) and tensile (sigmoid notch) strain lines provides superior stability; FEM confirmed biomechanical basis for non-parallel plate positioning
[21]	Lauer, 2007	Germany	Condylar neck	Delta plate	Titanium	Delta plate mimics two-plate function; suitable for endoscopic approach
[22]	Jung, 2020	Korea	Condylar base	Miniplates	Ti, Mg, PLLA, HA-PLLA	Metal maintains stress at fixation; HA-PLLA less stress transfer
[23]	Xin, 2014	China	Condylar head	Positional screws (2)	Titanium	Type B condylar head fractures; two-screw osteosynthesis transmits stress through screws with minimal stress on fractured surfaces
[24]	de Jesus, 2014	Brazil	Condylar base	Two straight plates, lambda plate, trapezoidal plate	Titanium	Lambda plate showed most homogeneous stress distribution for both plate and bone; best single-plate option
[25]	Aquilina, 2014	Australia	Condylar base	Four plates: 1.5 mm X, rectangular, square; 2.0 mm straight	Titanium	1.5-mm X plate comparable to single 2.0-mm plate; rectangular and square plates not recommended
[26]	Conci, 2015	Brazil	Condylar base	Neck screw vs conventional	Titanium	Neck screw comparable to single-plate fixation; two-plate system superior; neck screw may serve as reduction tool
[27]	Conci, 2018	Brazil	Condylar base	Neck screw (overlaid vs welded plate)	Titanium	Neck screw provides adequate stability comparable to single-plate fixation; welded point connection to plate offers no additional benefit
[28]	Zieliński, 2019	Poland	Condylar neck	A-shape plate	Ti, Bioresorbable	Titanium superior to PLLA: 2× lower bone pressure and 10× lower displacement; PLLA fixation too flexible for condylar neck fractures without additional immobilization
[29]	Pavlychuk, 2020	Ukraine	Condylar head	2 screws vs. 2 screws + patient-specific reinforcement plate (PSRP)	Titanium	PSRP reduced cortical bone stress 2–10× and increased fixation rigidity 1.25–3× vs. conventional two-screw fixation
[30]	Liokatis, 2021	Germany	Condylar neck	Alpha, kappa, rhomboidal, trapezoidal plates	Titanium	Alpha and kappa plates superior under high functional loads (500 N); trapezoidal and rhomboidal plates not recommended for condylar neck fractures; all 4 plates adequate under low postoperative load (135 N)
[31]	Bu, 2022	China	Condylar head	Single bicortical screw, 2 bicortical screws, screw + plate combinations	Titanium	Two-bicortical-screw fixation was most stable with lowest displacement and stress; titanium plates alleviated lever action but did not significantly reduce displacement; single-screw configurations exceeded screw yield limit
[32]	Schönegg, 2022	Switzerland	Condylar head	2 vs 3 screws	Titanium	Two screws sufficient for condylar head osteosynthesis
[33]	Keskin Yalcin, 2023	Turkey	Condylar base	Trapezoidal plate	Ti, PLLA	Biomechanical comparison of titanium and PLLA trapezoidal plates
[34]	Banerjee, 2023	India	Condylar head	Two positional screws	Titanium	Influence of screw distance in type B condylar head fractures
[35]	Chen, 2024	Taiwan	Condylar neck	Single vs double plate	Titanium	Comparable clinical outcomes; double plate biomechanically superior
[36]	Li, 2024	China	Condylar base (associated mandibular fractures)	Various RIF modalities	Titanium	Optimal fixation for condylar-base-associated multiple mandibular fractures
[37]	Liokatis, 2024	Germany	Condylar base	Trapezoidal plate	Titanium	How to achieve stable fixation at different fracture lines with trapezoid plate
[38]	Schönegg, 2024b	Switzerland	Condylar head	Screws (Ti, Mg, PLA)	Ti, Mg, PLA	Ti, Mg, PLA screw comparison; Ti best stability; Mg viable alternative; PLA unsuitable
[39]	Kumar, 2025	India	Condylar neck	5 plates: miniplate, lambda, trapezoidal, rhomboidal, alpha	Titanium	Alpha and lambda plates most stable with lowest hardware failure risk; trapezoidal plate least stable and most prone to failure for condylar neck fractures
[40]	Shouman, 2025	Egypt	Condylar base	Delta plate, double 2.0 miniplates	Titanium	Double 2.0 miniplates superior to delta plate (lower VM stress, bone stress, interfragmentary motion); delta plate acceptable when space is limited
[41]	Conci, 2025	Brazil	Condylar base	New device	Titanium	FEA supporting a new device for treating subcondylar fractures
[42]	Desai, 2025	India	Condylar base	Posterior border osteosynthesis	Titanium	Posterior border approach with FEA model corroboration
[43]	Dhirawani, 2025	India	Condylar base (bifocal, with contralateral fracture)	Rigid vs non-rigid	Titanium	Non-rigid fixation (single miniplate + arch bar) sufficient for bifocal mandibular fractures involving subcondylar and contralateral dentate fractures
[44]	Murakami, 2017	Japan	Condylar base	Various plates (1 Ti, 2 Ti, 2 PLLA, condylar plates)	Ti, PLLA	CT-based patient-specific FEA with heterogeneous bone properties; 5-hole strut plate optimal among condylar plates; 1 Ti plate and 2 PLLA plates exceeded compressive strength

Abbreviations: Ti = Titanium; u-HA/PLLA = unsintered hydroxyapatite/poly-L-lactic acid; Mg = Magnesium; PLLA = poly-L-lactic acid; HA-PLLA = hydroxyapatite/poly-L-lactic acid; LMOL = contralateral molar clench; MEF = Mechanical Excellence Factor; ORIF = open reduction internal fixation; CT = computed tomography; FEA = finite element analysis Fracture types follow the Loukota et al. classification (condylar head, condylar neck, condylar base); fractures previously described as subcondylar correspond to the condylar base level

### Temporal trends in research output

Analysis of the years of publication revealed distinct phases in FEA research for condylar fracture fixation. The foundational period (2002–2010) was characterized by methodological development and validation studies, with Wagner et al. [8] establishing fundamental modeling approaches. The expansion phase (2011–2019) witnessed increased research output with focus on comparative plate designs, as exemplified by studies from Syrian [14, 15], Japanese [12, 44], and Brazilian [24, 26, 27] research groups. The recent acceleration phase (2020–2025) has seen the highest publication rate, with 20 of 36 studies (56%) being published within this 5-year period. This recent surge reflects growing interest in patient-specific modeling, novel materials-including magnesium alloys, polylactic acid (PLA), and hydroxyapatite/poly-L-lactic acid (HA-PLLA) composites [19, 22, 38]and complex fracture scenarios [36, 43]. The geographical distribution of research has also expanded, with contributions from emerging centers in Korea [22], Turkey [33], Egypt [40], and Ukraine [29] joining those from established research centers.

### FEA methodology across the included studies

#### Model construction

Most included studies utilized high-resolution computed tomography (CT) data for 3D model construction, with a notable exception being the study by Parascandolo et al. [20], who developed their mandibular finite element method (FEM) from a real surface mock-up using virtual reality modeling language (VRML) triangulation. Common software platforms used included Mimics (Materialise, Belgium), Geomagic (3D Systems, USA), and SolidWorks (Dassault Systèmes, France). Models were analyzed using ANSYS Workbench (ANSYS Inc., USA), Mechanical Finder (RCCM, Japan), or Abaqus (Dassault Systèmes, France). The models were discretized using tetrahedral elements, with the number of elements ranging from approximately 464,000 to 1,500,000 and nodes from approximately 300,000 to 1,095,000; Aquilina et al. [1] specifically employed four-node linear tetrahedral (tet4) elements, and all three studies performed mesh convergence testing to ensure solution accuracy [1, 6, 12].

#### Material properties

The standard material properties used across the studies included cortical bone (elastic modulus [E] = 13,700–14,800 MPa, Poisson’s ratio [ν] = 0.30), cancellous bone (E = 1370–1850 MPa, ν = 0.30), and titanium (pure titanium and titanium alloy; E = 105,000–114,000 MPa, ν = 0.19–0.34) [5, 6, 44]. Murakami et al. [44] employed distinct material parameters for pure titanium grade 4 plates (E = 105,900 MPa, ν = 0.19) and Ti-6Al-7Nb alloy screws (E = 108,900 MPa, ν = 0.28), an approach subsequently adopted by Li et al. [6]. Most studies assumed homogeneous, isotropic, and linearly elastic material behavior, although earlier work by Hart et al. [4] demonstrated transversely isotropic properties for mandibular cortical bone, and Wagner et al. [8] employed patient-specific heterogeneous properties derived from CT Hounsfield unit values. A systematic review by Merema et al. [45] confirmed this lack of consensus, reporting Young’s modulus values for cortical bone ranging from 13.7 to 17 GPa across validated FEA models. Notable exceptions include Murakami et al. [44], who computed element-specific mechanical properties from CT Hounsfield unit values using the equations of Keyak et al. [46].; Albogha et al. [12] who incorporated patient-specific heterogeneous bone properties derived from CT Hounsfield Unit values; Gupta et al. [13] who utilized region-specific orthotropic material properties; Xin et al. [23] who assigned ten discrete material properties derived from CT gray values using default empirical formulas in Mimics; and Parascandolo et al. [20] who modeled anisotropic mandibular bone with regionally variable stiffness increasing from the posterior to the anterior mandibular body.

#### Boundary conditions

The boundary condition setup varied across studies, depending on how condylar loading was simulated. Most studies fixed all degrees of freedom at the condylar surface to simulate articulation with the glenoid fossa and applied masticatory forces at the occlusal surfaces [6, 14]. Aquilina et al. [1] employed a jaw-hinge mechanism that allowed rotational degrees of freedom along the condylar axis, with constraints being applied at the third molars to simulate unilateral biting. In contrast, Albogha et al. [12] applied a 150-N load directly to the condylar head with constraints at the molar region, representing a different modeling approach for condylar reaction forces. The contact interface between plate and bone was typically modeled as bonded (fully constrained) or frictional, with friction coefficients ranging from 0.3 to 0.5 for titanium–bone interfaces. Additionally, the contact between fractured bone surfaces was modeled as frictional rather than bonded in some studies; for example, Li et al. [6] assigned a friction coefficient of 0.46 to the interface between bone fragments, reflecting the rough surface interaction at the fracture site.

#### Loading conditions

Loading configurations simulated various masticatory tasks described in the work of Korioth and Hannam [47], including incisal clench, intercuspal position, ipsilateral molar clench, contralateral molar clench, and group function movements [6, 15]. Hijazi et al. [15] systematically analyzed different loading conditions and demonstrated that contralateral molar clench produces the highest stress concentrations in the fractured condylar region.

### Condylar head fracture studies

Recent years have seen increased attention being paid to condylar head fractures, which were previously underrepresented in the literature. Xin et al. [23] were the first to apply FEA to evaluate type B condylar head fractures secured using two positional screws. They demonstrated that stress was effectively transmitted through the screws to the medial fragments while stresses on the fractured sagittal surfaces remained minimal. This work was extended by Banerjee et al. [34], who investigated the influence of screw distance in two-screw osteosynthesis constructs. Bu et al. [31] compared four fixation configurations for condylar head fractures-single bicortical screw, two bicortical screws, one bicortical screw with one monocortical positional screw via a titanium plate, and two bicortical screws via a titanium plate-and demonstrated that fixation with two bicortical screws produced the smallest displacement and lowest stress on both implant materials and surrounding bone, whereas titanium plates further alleviated lever action but did not significantly reduce displacement. Schönegg et al. [32] compared two versus three screws for condylar head osteosynthesis, and found that two screws provided sufficient stability. Pavlychuk et al. [29] evaluated the biomechanical benefit of reinforcing conventional two-screw fixation of type p condylar head fractures with a patient-specific reinforcement plate, demonstrating that it provided a 2–10-fold reduction in cortical bone stress and 1.25–3-fold increase in fixation rigidity. Most recently, Schönegg et al. [19, 38] expanded the field by evaluating alternative screw materials including magnesium and PLA for condylar head fixation.

### Comparison between European studies and those from other regions

Table 2 summarizes the key differences between European studies and those from other regions. European studies (*n* = 12) predominantly focused on development of novel plate designs and mechanical testing protocols. Swiss researchers contributed extensively to condylar head fracture research, with multiple studies on screw osteosynthesis [19, 32]. German researchers developed the delta-shaped 3D plate for endoscopic-assisted osteosynthesis [21], evaluated the lambda plate for different condylar fracture patterns [18], and investigated the trapezoidal plate’s fixation rigidity at different fracture lines in the condylar base [37]. Asian studies and those from other regions (*n* = 24), including the Middle East, South America, and Africa, prioritized patient-specific modeling [12, 44], systematic comparison of plate geometries [12, 14], clinical applicability with particular attention to bioabsorbable materials [6, 22, 33, 34, 44], and novel device development [32].

**Table 2:** Comparison of the characteristics of European research with that from other regions

Aspect	European Studies (n = 12)	Other Nationality Studies (n = 24)
**Countries/Regions**	Austria (1), Germany (4), Italy (1), Poland (2), Switzerland (3), Ukraine (1)	Asia: Japan (2), China (4), Taiwan (2), Korea (1), India (5), Syria (2) South America: Brazil (4) Oceania: Australia (2) Middle East/Africa: Turkey (1), Egypt (1)
**Primary Research Focus**	• Novel 3D plate design and development • Condylar head fracture osteosynthesis • Mechanical testing protocols • Emerging materials (Mg screws) • Novel device development	• Patient-specific FEA modeling • Systematic plate geometry comparisons • Material comparisons (Ti vs bioabsorbable) • Loading condition analysis
**Plate Design Contributions**	• Delta plate (Germany) [21] • A-shape plate (Poland) [17, 28] • Lambda plate (Germany) [18] • Trapezoidal plate analysis (Germany) [37]	• Trapezoid plate validation (Syria, Japan) [12, 14] • Position A+B configuration (China) [6] • Neck screw technique (Brazil) [26] • New subcondylar device (Brazil) [41] • Posterior border approach (India) [42]
**Materials Evaluated**	Primarily titanium (*n* = 10) Magnesium screws (*n* = 2) [19, 38] Ti vs bioresorbable (*n* = 1) [28]	Titanium (*n* = 22) Ti vs u-HA/PLLA (*n* = 1) [6] Ti vs PLLA (*n* = 1) [33] Ti vs Mg alloy vs PLLA vs HA-PLLA (*n* = 1) [22]
**Fracture Types Studied**	Condylar neck (*n* = 4) Condylar head (*n* = 4) Condylar neck/base (*n* = 1) Subcondylar (*n* = 1) Condylar process (*n* = 1) Condylar base (*n* = 1)	Subcondylar (*n* = 10) Condylar neck (*n* = 4) Condylar head (*n* = 3) Condylar fracture (*n* = 2) Condylar base (*n* = 2) Complex/multiple (*n* = 5)

Ti = titanium; u-HA/PLLA = unsintered hydroxyapatite/poly-L-lactic acid; 3D = three dimensional; FEA = finite element analysis

#### Key findings and consensus

Despite methodological heterogeneity, several consensus findings have emerged. Across all studies, double-plate fixation consistently demonstrated superior biomechanical stability compared with single-plate configurations [1, 6, 16, 20, 35, 44]. Chen et al. [35] demonstrated comparable clinical outcomes between single long-plate and double short-plate fixations, with biomechanical simulations showing superior stability for the double-plate configuration. Three-dimensional plates (delta, trapezoidal) were shown to provide stability comparable to double miniplates [14, 21]. Among 3D plate designs, the trapezoid miniplate demonstrated the best overall performance for subcondylar and condylar base fractures, achieving the greatest rigidity with the lowest bone strains, as independently reported by Syrian [14] and Japanese [12] research groups. Liokatis et al. [37] further demonstrated that for condylar base fractures, the rigidity of the trapezoidal plate’s fixation depends on the fracture line position and plate placement, with more cranial positioning providing better stabilization for lower base fractures. However, this favorable performance may not extend to condylar neck fractures; Kumar et al. [39] demonstrated that among five plate designs evaluated for condylar neck fracture fixation, the trapezoidal plate was the least stable and most prone to hardware failure, with alpha and lambda plates showing superior performance. Liokatis et al. [18] further demonstrated that the lambda plate provides adequate fixation rigidity for condylar neck fractures, particularly when placed as cranially as possible. However, the lambda plate did not achieve sufficient stability for condylar base fractures, for which they recommended supplementation with a second plate at the sigmoid notch. Notably, these findings partially contrast with those of de Jesus et al. [24], who reported that among three fixation methods evaluated, the lambda plate showed the most homogeneous stress distribution for condylar base fractures. This discrepancy between the two studies may reflect differences in fracture modeling, plate positioning, and evaluation criteria. Additionally, Murakami et al. [44] compared box, strut, and lambda condylar plates and found that the five-hole strut plate produced the smallest tensile and compressive stresses, likely because its cross bars effectively reduced stress concentration; this computational finding was clinically validated in the same patient with successful bone healing at 6-month follow-up.

Three key implanting positions have been identified on the mandibular condylar region: Position A along the posterolateral border of the ramus; Position B along the anterolateral border following the sigmoid notch curvature; and Position C at the lateral border in the middle area. Of these, the A+B configuration was shown to provide optimal biomechanical performance for both titanium and bioabsorbable materials [6]. The contralateral molar clenching loading condition was identified as producing the highest stress in condylar osteosynthesis components [6, 13, 15], and Huang et al. [16] evaluated subcondylar fracture fixation under this condition, demonstrating that posterior miniplate placement provides greater stability with less sliding distance in comparison with anterolateral placement.

Regarding plate placement for bioabsorbable fixation, Li et al. [6] demonstrated that the A+B configuration, which has been identified as optimal for titanium, also provides sufficient stability for condylar base fracture treatment with u-HA/PLLA plates. However, Murakami et al. [44] did not recommend PLLA plates for subcondylar fractures, even with double-plate fixation, because compressive stresses exceeded the compressive strength of PLLA. This finding highlights the fact that the suitability of bioabsorbable materials depends on both the specific polymer composition and the fracture configuration. In vitro biomechanical testing by Sukegawa et al. [2] further supports these findings through its demonstration that although u-HA/PLLA plates possess only approximately 45% of the tensile and shear strength of titanium plates, their resistance is adequate in double-plate fixation configurations. Jung et al. [22] compared four fixation materials (titanium, magnesium alloy, PLLA, and HA-PLLA) for subcondylar fracture and demonstrated that HA-PLLA showed less stress transfer to biologic components than did PLLA, while maintaining deformation within clinically acceptable limits, suggesting its potential as a biodegradable alternative to metal fixation. For condylar head fractures, two-screw osteosynthesis has been established as the standard approach [23, 32, 34], with the influence of screw positioning and distance having been systematically evaluated [34]. Emerging materials such as magnesium alloys have shown favorable biomechanical behavior with fragment deformation and fracture displacement properties similar to those of titanium, although stress values may exceed their tensile strength under extreme loading conditions. Therefore, magnesium screws may represent a viable alternative to titanium in selected cases [19, 38], whereas PLA screws are unsuitable for condylar head osteosynthesis because of insufficiencies in their mechanical properties [38].

Recent studies have also addressed complex clinical scenarios including bifocal mandibular fractures [43], condylar-base-associated multiple mandibular fractures [36], and posterior border osteosynthesis approaches [42].

## Discussion

This scoping review mapped 36 studies investigating FEA-based internal fixation of mandibular condylar fractures, revealing a field that has undergone rapid maturation, with 56% of all studies being published since 2020. The distinct research trajectories observed between European and other regions likely reflect differences in academic–industrial partnerships and clinical priorities: European centers, benefiting from close collaboration with implant manufacturers, have focused on iterative refinement of novel plate designs, whereas research groups from Asia, South America, and other regions, often operating in settings where cost-effectiveness and material alternatives are of greater concern, have emphasized systematic comparisons across existing platforms and bioabsorbable materials. Rather than representing a limitation, this complementarity has strengthened the collective evidence base by generating converging conclusions from fundamentally different methodological perspectives.

The methodological approaches identified in this review align with broader trends in applying FEA in maxillofacial research [5]. Similar heterogeneity in modeling assumptions and boundary conditions has been observed in FEA studies of mandibular angle fractures [48] and orthognathic surgery [49], and FEA has also been applied to analyze buttress biomechanics in the midface [50]. The predominant use of homogeneous isotropic material properties in condylar fracture studies mirrors the practices in other maxillofacial applications, despite evidence that bone exhibits anisotropic and heterogeneous properties [45]. In this respect, Merema et al. reported that mandibular cortical bone properties vary according to anatomical location and exhibit direction-dependent mechanical behavior, although their review revealed no consensus on how to incorporate these characteristics into FEA models. While heterogeneous property assignment based on CT gray values represents a more realistic approach, it requires patient-specific calibration and standardized protocols that are currently lacking. This methodological limitation is not unique to condylar fracture research but reflects broader challenges in computational biomechanics, where simplifying assumptions are often necessary to maintain computational feasibility.

Several converging patterns emerged across the mapped studies. The superiority of the A+B double-plate configuration and the fracture-site dependence of 3D-plate performance–favorable for the trapezoid plate at the subcondylar and condylar base levels but less so at the condylar neck, where alpha and lambda plates performed better–were reported consistently by independent groups [12, 14, 18, 20, 37, 39]. A detailed mechanistic interpretation of these patterns is beyond the scope of a scoping review; we therefore present them as mapped evidence and refer readers to the primary biomechanical studies for the underlying strain-distribution rationale.

The strength of these computational predictions is reinforced by converging evidence from independent experimental methods. In vitro biomechanical testing by Tominaga et al. [51] demonstrated that double adaptation plate fixation showed approximately five times greater load-bearing capacity than single plating in synthetic mandible models, providing complementary experimental evidence to support the FEA predictions. Furthermore, using a porcine mandible cadaver model, Haim et al. [52] confirmed that the Delta plate and the TriLock Delta condyle trauma plate provide functional stability comparable to dual miniplate osteosynthesis, with the locking plate demonstrating greater primary stability and decreased likelihood of screw loosening. Kozakiewicz et al. [53] systematically evaluated 51 plate designs for condylar base fractures using in vitro testing and proposed the Mechanical Excellence Factor as a predictive tool for plate fixation rigidity, with their findings: that double plates provide the highest rigidity and plates with 7–10 screws and transverse reinforcing bars achieve optimal performance and therebydirectly complementing the FEA predictions. This multi-method triangulation across computational, synthetic model, cadaveric, and clinical investigations substantially strengthens the evidence base and lends credibility to the broader FEA-derived conclusions of this review. Similarly, the in vitro validation of bioabsorbable materials for condylar fracture fixation [2, 6, 22] expands treatment options, particularly for pediatric patients or those requiring postoperative imaging surveillance without metallic artifact interference.

Despite robust biomechanical evidence, clinical decision-making must account for factors that current FEA models do not capture, including surgical access limitations, operative time, cost, and patient-specific variables such as bone quality and fracture comminution. The fracture-site dependence of plate performance has direct implications for surgical planning: while the trapezoid plate may offer an acceptable single-plate alternative for subcondylar fractures where dual-plate fixation is technically challenging, such as cases requiring a transoral approach [54], clinical data from Smolka et al. [55] confirming higher rates of plate and screw loosening at the condylar neck particularly with major fragment displacement and contact loss validate the computational predictions of Kumar et al. [39] and underscore the importance of fracture-level assessment in plate selection. This convergence of FEA predictions with clinical observations represents a compelling example of how computational and clinical evidence can be integrated to guide fixation strategy. For condylar head fractures, long-term clinical follow-up demonstrating stable functional and anatomical outcomes with small-fragment positional screw osteosynthesis using two to three screws [56] further supports the practical value of minimizing hardware in anatomically constrained surgical fields. This biomechanical equivalence is mirrored in the available clinical literature. A recent systematic review and meta-analysis by Kuna et al. [57] found no significant differences in operative time, maximum mouth opening, complication rates, or functional outcomes between fixation with two miniplates and fixation with a three-dimensional plate for mandibular condylar fractures, and a randomized clinical study by Ahuja et al. [58] similarly reported no significant difference in clinical outcomes between delta (three-dimensional) plates and conventional miniplates. Taken together with the present FEA findings, these data indicate that trapezoidal or three-dimensional single-plate fixation may achieve clinical outcomes comparable to those of the two-miniplate A+B configuration for condylar base fractures, supporting it as a clinically relevant alternative when dual-plate fixation is technically challenging.

Several promising research directions emerge from this review. A critical limitation of current FEA studies is the reliance on idealized or single-patient models that fail to capture the heterogeneity encountered in clinical practice. Mandibular condylar fractures present with considerable anatomical and morphological variations, as reflected in the established classification systems [3], including differences in fracture line orientation, fragment displacement patterns, condylar head morphology, and bone density distribution. Future studies incorporating multiple patient-derived models representing the diverse spectrum of fracture configurations observed in actual clinical populations would significantly enhance the clinical applicability of FEA predictions. Such patient-specific variation-focused approaches, building upon the heterogeneous bone modeling methodology employed by Murakami et al. [44] and Albogha et al. [12], could establish more nuanced fixation recommendations tailored to specific fracture subtypes, rather than generalized guidelines.

The development of standardized FEA protocols also represents an immediate priority; consensus guidelines on mesh density thresholds, material property databases, and loading condition specifications would enhance reproducibility and enable quantitative comparisons across studies. The current lack of standardization, which is evident in the wide ranges of reported material properties and the predominance of homogeneous isotropic assumptions despite known anisotropic bone behavior [45], limits the ability to perform meaningful meta-analyses and may contribute to the discrepancies observed between studies evaluating the same fixation methods, such as the conflicting reports on lambda plate performance for condylar base fractures from Liokatis et al. [18] and de Jesus et al. [24]. The emergence of magnesium-based and other biodegradable implants [19, 38] presents an exciting frontier, although long-term in vivo studies evaluating degradation kinetics and effects on bone remodeling remain essential before widespread clinical adoption. Additionally, although condylar head fracture research has shown substantial expansion, prospective clinical studies correlating FEA predictions with functional outcomes are critically needed to validate computational models and establish their role in personalized surgical planning.

### Limitations

This scoping review has several limitations. First, the search was restricted to PubMed and the Cochrane Library and to English-language publications, so relevant studies in other databases or languages may have been missed. Second, in accordance with scoping-review methodology, we did not perform a formal critical appraisal or risk-of-bias assessment of the included studies; the reliability of individual findings was therefore not graded. Third, the marked heterogeneity in modeling assumptions, material properties, boundary and loading conditions, and outcome measures precluded any quantitative synthesis, so the consensus statements reflect qualitative convergence rather than pooled estimates. Fourth, all included studies were computational; inherent FEA simplifications–such as idealized geometry, homogeneous isotropic material assumptions, and reliance on single-patient models–limit direct extrapolation to clinical practice. Finally, the geographic comparison was exploratory and descriptive and is constrained by the small number and uneven distribution of studies. These limitations should be considered when interpreting the mapped findings.

## Conclusion

This scoping review provides a comprehensive map of FEA research investigating the internal fixation of mandibular condylar fractures. Thirty-six studies from European, Asian, South American, Middle Eastern, and African research centers were identified, with these revealing distinct but complementary research trajectories. Across the FEA evidence mapped in this review, double-plate fixation in the A+B configuration was the most consistently favorable approach for both titanium and bioabsorbable systems, with the trapezoid plate demonstrating the best performance among single 3D plate alternatives for subcondylar and condylar base fractures, although its efficacy may be limited for condylar neck fractures, for which alpha and lambda plates have shown superior stability. For condylar head fractures, two-screw osteosynthesis has been shown to be effective, with magnesium emerging as a promising alternative material, but PLA being found to be unsuitable. Future research should prioritize standardized FEA protocols, patient-specific modeling, clinical outcome validation, and evaluation of emerging materials to further advance evidence-based management of mandibular condylar fractures.

## Electronic supplementary material

Below is the link to the electronic supplementary material.

Supplementary Material 1

## Data Availability

All data analyzed during this study are included in this published article and its tables.

## References

[1] Aquilina P, Chamoli U, Parr WCH, Clausen PD, Wroe S. Finite element analysis of three patterns of internal fixation of fractures of the mandibular condyle. Br J Oral Maxillofac Surg. 2013;51(4):326–31. 10.1016/j.bjoms.2012.08.007. 22981343 10.1016/j.bjoms.2012.08.007

[2] Sukegawa S, Kanno T, Yamamoto N, Nakano K, Takabatake K, Kawai H, et al. Biomechanical loading comparison between titanium and unsintered hydroxyapatite/poly-L-lactide plate system for fixation of mandibular subcondylar fractures. Materials. 2019;12(9):1557. 10.3390/ma12091557. 31085981 10.3390/ma12091557 PMC6539901

[3] Loukota RA, Eckelt U, De Bont L, Rasse M. Subclassification of fractures of the condylar process of the mandible. Br J Oral Maxillofac Surg. 2005;43(1):72–73. 10.1016/j.bjoms.2004.08.018. 15620780 10.1016/j.bjoms.2004.08.018

[4] Hart RT, Hennebel VV VV, Thongpreda N, Van Buskirk WC, RC. Modeling the biomechanics of the mandible: a three-dimensional finite element study. J Biomech. 1992;25(3):261–86. 10.1016/0021-9290(92)90025-V. 10.1016/0021-9290(92)90025-v 1564061

[5] Lisiak-Myszke M, Marciniak D, Bieliński M, Sobczak H, Garbacewicz Ł, Drogoszewska B. Application of finite element analysis in Oral and Maxillofacial Surgery—A literature review. Materials. 2020;13(14):3063. 10.3390/ma13143063. 32659947 10.3390/ma13143063 PMC7411758

[6] Li J, Jiao J, Luo T, Wu W. Biomechanical evaluation of various internal fixation patterns for unilateral mandibular condylar base fractures: a three-dimensional finite element analysis. J Mech Behav Biomed Mater. 2022;133:105354. 10.1016/j.jmbbm.2022.105354. 35793604 10.1016/j.jmbbm.2022.105354

[7] Li J, Yang H, Han L. Open versus closed treatment for unilateral mandibular extra-capsular condylar fractures: a meta-analysis. J Cranio-Maxillofacial Surg. 2019;47(7):1110–19. 10.1016/j.jcms.2019.03.021. 10.1016/j.jcms.2019.03.021 30962040

[8] Wagner A, Krach W, Schicho K, Undt G, Ploder O, Ewers R. A 3-dimensional finite-element analysis investigating the biomechanical behavior of the mandible and plate osteosynthesis in cases of fractures of the condylar process. Oral Surg, Oral Med, Oral Pathol, Oral Radiol, Endodontology. 2002;94(6):678–86. 10.1067/moe.2002.126451. 10.1067/moe.2002.126451 12464890

[9] García-Guerreroa I, Ramírezb JM, Gómez de Diego R, Martínez-Gonzálezd JM, Poblador MS, Lancho JL. Complications in the treatment of mandibular condylar fractures: surgical versus conservative treatment. Ann Anat - Anatomischer Anz. 2018;216:60–68. 10.1016/j.aanat.2017.10.007. 10.1016/j.aanat.2017.10.007 29223659

[10] Peters MDJ, Godfrey CM, Khalil H, McInerney P, Parker D, Soares CB. Guidance for conducting systematic scoping reviews. Int J Evidence-Based Healthcare. 2015;13(3):141–46. 10.1097/XEB.0000000000000050. 10.1097/XEB.0000000000000050 26134548

[11] Tricco AC, Lillie E, Zarin W, O’Brien KK, Colquhoun H, Levac D, et al. PRISMA extension for scoping reviews (PRISMA-ScR): checklist and explanation. Ann Intern Med. 2018;169(7):467–73. 10.7326/M18-0850. 30178033 10.7326/M18-0850

[12] Albogha MH, Mori Y, Takahashi I. Three-dimensional titanium miniplates for fixation of subcondylar mandibular fractures: comparison of five designs using patient-specific finite element analysis. J Cranio-Maxillofacial Surg. 2018;46(3):391–97. 10.1016/j.jcms.2017.12.020. 10.1016/j.jcms.2017.12.020 29311022

[13] Gupta A, Dutta A, Dutta K, Mukherjee K. Biomechanical influence of plate configurations on mandible subcondylar fracture fixation: a finite element study. Med Biol Eng Comput. 2023;61(10):2581–91. 10.1007/s11517-023-02854-7. 37233860 10.1007/s11517-023-02854-7

[14] Darwich MA, Albogha MH, Abdelmajeed A, Darwich K. Assessment of the biomechanical performance of 5 plating techniques in fixation of mandibular subcondylar fracture using finite element analysis. J Oral Maxillofacial Surg. 2016;74(4):.e794.1–48. 10.1016/j.joms.2015.11.021. 10.1016/j.joms.2015.11.021 26706490

[15] Hijazi L, Hejazi W, Darwich MA, Darwich K. Finite element analysis of stress distribution on the mandible and condylar fracture osteosynthesis during various clenching tasks. Oral Maxillofac Surg. 2016;20(4):359–67. 10.1007/s10006-016-0573-2. 27663241 10.1007/s10006-016-0573-2

[16] Huang CM, Chan MY, Hsu JT, Su KC. Biomechanical analysis of subcondylar fracture fixation using miniplates at different positions and of different lengths. BMC Oral Health. 2021;21(1):543. 10.1186/s12903-021-01905-5. 34674692 10.1186/s12903-021-01905-5 PMC8532336

[17] Kozakiewicz M, Swiniarski J. “A” shape plate for open rigid internal fixation of mandible condyle neck fracture. J Cranio-Maxillofacial Surg. 2014;42(6):730–37. 10.1016/j.jcms.2013.11.003. 10.1016/j.jcms.2013.11.003 24359864

[18] Liokatis P, Tzortzinis G, Gerasimidis S, Smolka W. Application of the lambda plate on condylar fractures: finite element evaluation of the fixation rigidity for different fracture patterns and plate placements. Injury. 2022;53(4):1345–52. 10.1016/j.injury.2022.01.032. 35101256 10.1016/j.injury.2022.01.032

[19] Schönegg D, Müller GT, Blumer M, Essig H, Wagner MEH. Two versus three magnesium screws for osteosynthesis of mandibular condylar head fractures: a finite element analysis. Clin Oral Investig. 2024;28(10):553. 10.1007/s00784-024-05927-5. 39327352 10.1007/s00784-024-05927-5 PMC11427473

[20] Parascandolo S, Spinzia A, Parascandolo S, Piombino P, Califano L. Two load sharing plates fixation in mandibular condylar fractures: biomechanical basis. J Cranio-Maxillofacial Surg. 2010;38(5):385–90. 10.1016/j.jcms.2009.10.014. 10.1016/j.jcms.2009.10.014 19944616

[21] Lauer G, Pradel W, Schneider M, Eckelt U. A new 3-dimensional plate for transoral endoscopic-assisted osteosynthesis of condylar neck fractures. J Oral Maxillofacial Surg. 2007;65(5):964–71. 10.1016/j.joms.2006.05.068. 10.1016/j.joms.2006.05.068 17448849

[22] Jung BT, Kim WH, Park B, Lee J-H, Kim B, Lee J-H, et al. Biomechanical evaluation of unilateral subcondylar fracture of the mandible on the varying materials: a finite element analysis. PLoS One. 2020;15(10):e0240352. 10.1371/journal.pone.0240352. 10.1371/journal.pone.0240352 PMC7544122 33031474

[23] Xin P, Jiang B, Dai J, Hu G, Wang X, Xu B, et al. Finite element analysis of type B condylar head fractures and osteosynthesis using two positional screws. J Cranio-Maxillofacial Surg. 2014;42(5):482–88. 10.1016/j.jcms.2013.06.006. 10.1016/j.jcms.2013.06.006 23906675

[24] de Jesus GP, Vaz LG, Gabrielli MFR, Passeri LA, Oliveira TV, Noritomi PY, et al. Finite element evaluation of three methods of stable fixation of condyle base fractures. Int J Oral Max Surg. 2014;43(10):1251–56. 10.1016/j.ijom.2014.07.011. 10.1016/j.ijom.2014.07.011 25124390

[25] Aquilina P, Parr WCH, Chamoli U, Wroe S, Clausen P. A biomechanical comparison of three 1.5-mm plate and screw configurations and a single 2.0-mm plate for internal fixation of a mandibular condylar fracture. Craniomaxillofacial Trauma Reconstr. 2014;7(3):218–23. 10.1055/s-0034-1375172. 10.1055/s-0034-1375172 PMC4130755 25136411

[26] Conci RA, Tomazi FHS, Noritomi PY, da Silva JVL, Fritscher GG, Heitz C. Comparison of neck screw and conventional fixation techniques in mandibular condyle fractures using 3-dimensional finite element analysis. J Oral Maxillofacial Surg. 2015;73(7):1321–27. 10.1016/j.joms.2015.01.037. 10.1016/j.joms.2015.01.037 25869984

[27] Conci RA, Garbin E Jr, Griza GL, Érnica NM, Noritomi PY, Tomazi FHS, et al. Does lag screw fixation of condylar fractures result in adequate stability? A finite element analysis. J Cranio-Maxillofacial Surg. 2018;46(6):1041–45. 10.1016/j.jcms.2018.04.008. 10.1016/j.jcms.2018.04.008 29735385

[28] Zieliński R, Kozakiewicz M, Swiniarski J. Comparison of Titanium and bioresorbable plates in “A” shape plate properties—finite element analysis. Materials. 2019;12(7):1110. 10.3390/ma12071110. 30987137 10.3390/ma12071110 PMC6480357

[29] Pavlychuk T, Chernogorskyi D, Chepurnyi Y, Neff A, Kopchak A. Biomechanical evaluation of type p condylar head osteosynthesis using conventional small-fragment screws reinforced by a patient specific two-component plate. Head Face Med. 2020;16(1):25. 10.1186/s13005-020-00236-0. 33076933 10.1186/s13005-020-00236-0 PMC7574441

[30] Liokatis P, Tzortzinis G, Gerasimidis S, Smolka W. Finite element analysis of different titanium plates for internal fixation of fractures of the mandibular condylar neck. J Oral Maxillofacial Surg. 2021;79(3):.e665.1–665.10. 10.1016/j.joms.2020.09.038. 10.1016/j.joms.2020.09.038 33148415

[31] Bu L, Chen Q, Huang K, Zhao X, Zheng J, Qiu Y, et al. Evaluation of internal fixation techniques for condylar head fractures: a finite element analysis and comparison. Oral Surg Oral Med Oral Pathol Oral Radiol. 2022;133(5):e96–104. 10.1016/j.oooo.2021.08.028. 10.1016/j.oooo.2021.08.028 34716116

[32] Schönegg D, Müller GT, Blumer M, Essig H, Wagner MEH. Two- versus three-screw osteosynthesis of the mandibular condylar head: a finite element analysis. J Mech Behav Biomed Mater. 2022;127:105077. 10.1016/j.jmbbm.2022.105077. 35033984 10.1016/j.jmbbm.2022.105077

[33] Yalcin BK. Biomechanical comparison of titanium and poly-L-lactic acid trapezoidal plates applied in a subcondylar fracture model. J Craniofac Surg. 2023;34(6):1737–40. 10.1097/SCS.0000000000009238. 36856431 10.1097/SCS.0000000000009238 PMC10445633

[34] Banerjee A, Rana M, Chakraborty A, Biswas JK, Chowdhury AR. In-silico study of type ‘B’ condylar head fractures and evaluating the influence of two positional screw distance in two-screw osteosynthesis construct. Proc Inst Mech Eng H. 2023;237(11):1297–305. 10.1177/09544119231201782. 37924244 10.1177/09544119231201782

[35] Chen CC, Chiu TH, Yan CY, Hou YP, Lin TS. Single versus double plate fixation in condylar neck fractures: clinical results and biomechanics simulation. Bioengineering. 2024;11(7):704. 10.3390/bioengineering11070704. 39061786 10.3390/bioengineering11070704 PMC11273754

[36] Li J, Xu CT, Li Y, Liang Y, Wu W, Li CY. Biomechanical evaluation of various rigid internal fixation modalities for condylar-base-associated multiple mandibular fractures: a finite element analysis. Med Biol Eng Comput. 2024;62(9):2787–803. 10.1007/s11517-024-03102-2. 38698188 10.1007/s11517-024-03102-2

[37] Liokatis P, Tzortzinis G, Cornelius CP, Malenova Y, Obermeier KT, Smolka W. A finite element analysis of the trapezoidal plate. How to get a stable fixation at different fracture lines? Injury. 2024;55(12):112020. 10.1016/j.injury.2024.112020. 39549421 10.1016/j.injury.2024.112020

[38] Schönegg D, Koch A, Müller GT, Blumer M, Wagner MEH. Two-screw osteosynthesis of the mandibular condylar head with different screw materials: a finite element analysis. Comput Methods Biomech Biomed Eng. 2024;27(7):878–82. 10.1080/10255842.2023.2209247. 10.1080/10255842.2023.2209247 37154519

[39] Kumar AK, Christopher P, Afradh M, Shenoy V. Finite element analysis of titanium plates for unilateral condylar neck fracture fixation: a computational study. J Maxillofac Oral Surg. 2025;24(5):1245–55. 10.1007/s12663-025-02445-z. 41054451 10.1007/s12663-025-02445-z PMC12496311

[40] Shouman HA, Metwally KG, Elhadidi Y, Abd El Wahed H. Elhadidi Y, Abd El Wahed H. Finite element analysis of two patterns of internal fixation of the mandibular subcondylar fractures (comparative study). J Maxillofac Oral Surg. 2025;24(1):123–29. 10.1007/s12663-024-02420-0. 39902407 10.1007/s12663-024-02420-0 PMC11787104

[41] Conci RA, Zanin RF, de Oliveira Andriola F, Fritscher GG, Heitz C. Finite element analysis (FEA) to support a new device to treat mandible subcondylar fractures. J Craniofac Surg. 2025;36(8):e1473–7. 10.1097/SCS.0000000000012002. 10.1097/SCS.0000000000012002 41037791

[42] Desai N, Panneerselvam E, Arun Vignesh KR, Balasubramanian S, Raja VBKK. Posterior border osteosynthesis for fractures of mandibular condyle: a case series with finite element model corroboration. J Maxillofac Oral Surg. 2025;24(2):353–59. 10.1007/s12663-024-02190-9. 40182443 10.1007/s12663-024-02190-9 PMC11961821

[43] Dhirawani AD, Sidana S, Natarajan S, Agrawal R. Comparative evaluation of stability of rigid and non-rigid fixation in bifocal fractures of mandible involving a unilateral mandibular subcondyle fracture associated with a contralateral mandibular dentate fracture: a finite element analysis. J Maxillofac Oral Surg. 2025;24(6):1650–60. 10.1007/s12663-025-02744-5. 41306253 10.1007/s12663-025-02744-5 PMC12644290

[44] Murakami K, Yamamoto K, Sugiura T, Horita S, Matsusue Y, Kirita T. Computed tomography–based 3-dimensional finite element analyses of various types of plates placed for a virtually reduced unilateral condylar fracture of the mandible of a patient. J Oral Maxillofacial Surg. 2017;75(6):.e1239.1–1239.11. 10.1016/j.joms.2017.02.014. 10.1016/j.joms.2017.02.014 28325640

[45] Merema BBJ, Kraeima J, Glas HH, Spijkervet FKL, Witjes MJH. Patient-specific finite element models of the human mandible: lack of consensus on current set-ups. Oral Dis. 2021;27(1):42–51. 10.1111/odi.13381. 32372548 10.1111/odi.13381 PMC7818111

[46] Keyak JH, Rossi SA, Jones KA, Skinner HB. Prediction of femoral fracture load using automated finite element modeling. J Biomech. 1997;31(2):125–33. 10.1016/S0021-9290(97)00123-1. 10.1016/s0021-9290(97)00123-1 9593205

[47] Korioth TWP, Hannam AG. Deformation of the human mandible during simulated tooth clenching. J Dent Res. 1994;73(1):56–66. 10.1177/00220345940730010801. 8294619 10.1177/00220345940730010801

[48] Kimsal J, Baack B, Candelaria L, Khraishi T, Lovald S. Biomechanical analysis of mandibular angle fractures. J Oral Maxillofacial Surg. 2011;69(12):3010–14. 10.1016/j.joms.2010.12.042. 10.1016/j.joms.2010.12.042 21496988

[49] Takahashi H, Moriyama S, Furuta H, Matsunaga H, Sakamoto Y, Kikuta T. Three lateral osteotomy designs for bilateral sagittal split osteotomy: biomechanical evaluation with three-dimensional finite element analysis. Head Face Med. 2010;6(1):4. 10.1186/1746-160X-6-4. 20346142 10.1186/1746-160X-6-4 PMC2853503

[50] Crespo Reinoso P, Jerez Robalino J, González de Santiago M. Biomechanics of midface trauma: a review of concepts. J Oral Maxillofacial Surg, Med, Pathol. 2021;33(4):389–93. 10.1016/j.ajoms.2021.01.010.

[51] Tominaga K, Habu M, Khanal A, Mimori Y, Yoshioka I, Fukuda J. Biomechanical evaluation of different types of rigid internal fixation techniques for subcondylar fractures. J Oral Maxillofacial Surg. 2006;64(10):1510–16. 10.1016/j.joms.2006.03.038. 10.1016/j.joms.2006.03.038 16982310

[52] Haim D, Müller A, Leonhardt H, Nowak A, Richter G, Lauer G. Biomechanical study of the delta plate and the trilock delta condyle trauma plate. J Oral Maxillofacial Surg. 2011;69(10):2619–25. 10.1016/j.joms.2011.01.002. 10.1016/j.joms.2011.01.002 21474224

[53] Kozakiewicz M, Okulski J, Krasowski M, Konieczny B, Zieliński R. Which of 51 plate designs can most stably fixate the fragments in a fracture of the mandibular condyle base? JCM. 2023;12(13):4508. 10.3390/jcm12134508. 37445541 10.3390/jcm12134508 PMC10343011

[54] Blumer M, Guggenbühl T, Wagner MEH, Rostetter C, Rücker M, Gander T. Outcome of surgically treated fractures of the condylar process by an endoscopic assisted transoral approach. J Oral Maxillofacial Surg. 2019;77(1):.e133.1–39. 10.1016/j.joms.2018.08.013. 10.1016/j.joms.2018.08.013 30227123

[55] Smolka W, Liokatis P, Cornelius CP. Evaluation of Complications after open reduction and internal fixation of mandibular condylar base and neck fractures using trapezoidal plates. J Craniofac Surg. 2020;31(5):1287–90. 10.1097/SCS.0000000000006486. 32371715 10.1097/SCS.0000000000006486

[56] Kolk A, Neff A. Long-term results of ORIF of condylar head fractures of the mandible: a prospective 5-year follow-up study of small-fragment positional-screw osteosynthesis (SFPSO). J Cranio-Maxillofacial Surg. 2015;43(4):452–61. 10.1016/j.jcms.2015.02.004. 10.1016/j.jcms.2015.02.004 25773375

[57] Kuna SK, Jain A, Kuna V. Two miniplates versus three dimensional plate in management of mandibular condylar fractures: a systematic review and meta-analysis. Craniomaxillofacial Trauma Reconstr. 2024;17(4):NP332–44. 10.1177/19433875241252979. 10.1177/19433875241252979 PMC11562994 39553811

[58] Ahuja SA, Galinde J, Asnani U, Mistry YA. Comparative evaluation of clinical outcomes using delta plates and conventional miniplates for internal fixation of mandibular condylar fractures in adults. J Oral Maxillofacial Surg. 2018;76(6):1255–66. 10.1016/j.joms.2017.12.018. 10.1016/j.joms.2017.12.018 29360455

