# Supplementary material for: Three-dimensional finite element analysis for internal fixation of mandibular condylar fractures: a scoping review
Source: Eur J Trauma Emerg Surg. 2026 Jul 8;52(1):217. doi: 10.1007/s00068-026-03259-5 (PMC13346322; doi:10.1007/s00068-026-03259-5)
Supplement: Supplementary file 1 — Supplementary Material 1 [file 68_2026_3259_MOESM1_ESM.docx]

Supplementary Table S1. Standardized Data-Charting Form

Data-extraction instrument used by two independent reviewers for each included study. One form was completed per study; disagreements were resolved by discussion or by a third reviewer.

| 1. Study identification | |
| --- | --- |
| Record ID / reference no. | Internal ID and citation number |
| First author | Surname of first author |
| Year of publication | Year |
| Country / region of institution | Country of the institution where the study was performed (per author affiliation); region group: European / other regions |
| Study aim | Stated objective of the study |
| 2. Fracture characteristics | |
| Fracture type (Loukota) | Condylar head / condylar neck / condylar base (subcondylar = condylar base) |
| Fracture model / pattern | Side, displacement, comminution, bifocal or associated fractures, fracture-line orientation |
| 3. FEA methodology | |
| Imaging / geometry source | CT data, surface mock-up, or other |
| Modeling software | e.g., Mimics, Geomagic, SolidWorks |
| Analysis (solver) software | e.g., ANSYS, Abaqus, Mechanical Finder |
| Element type | e.g., tetrahedral (tet4), other |
| No. of elements / nodes | Reported counts (if available) |
| Mesh convergence test | Yes / No / not reported |
| Material model | Isotropic vs anisotropic; homogeneous vs heterogeneous (e.g., CT-Hounsfield-derived) |
| 4. Material properties | |
| Cortical bone | Young’s modulus E (MPa); Poisson’s ratio ν |
| Cancellous bone | Young’s modulus E (MPa); Poisson’s ratio ν |
| Implant material(s) | Material(s) (Ti, Ti-alloy, u-HA/PLLA, PLLA, HA-PLLA, Mg, PLA) with E and ν |
| 5. Boundary & contact conditions | |
| Constraints | Degrees of freedom fixed; constraint location (condyle, molars) |
| Condylar loading approach | Fixed condylar surface vs applied condylar reaction force vs jaw-hinge mechanism |
| Contact definitions | Plate–bone and fracture-interface contact (bonded / frictional); friction coefficient(s) |
| 6. Fixation systems evaluated | |
| Plate type / design | e.g., straight miniplate, trapezoid, delta, lambda, alpha, strut, box |
| Screw configuration | Number, type (mono-/bicortical, positional/lag), spacing |
| No. / configuration of plates | Single, double parallel, double offset, A+B, etc. |
| Plate position(s) | Position A (posterolateral ramus) / B (anterolateral sigmoid notch) / C (lateral mid) |
| 7. Loading conditions | |
| Masticatory task(s) | Incisal clench, intercuspal, ipsilateral/contralateral molar clench, group function |
| Applied force | Magnitude (N), direction, point of application |
| 8. Outcome measures | |
| Stress | von Mises / principal stress on bone and/or implant (location, magnitude) |
| Strain | Bone / interfragmentary strain |
| Displacement | Fragment / interfragmentary displacement; relative micromotion |
| Other outcomes | Fixation rigidity, factor of safety, MEF, deformation, etc. |
| 9. Findings & notes | |
| Key biomechanical finding(s) | Principal results relevant to the review questions |
| Authors’ conclusion | Authors’ stated conclusion / recommendation |
| Clinical / experimental validation | Any in vitro, cadaveric, or clinical corroboration reported |
| Reviewer notes | Discrepancies, ambiguities, or items requiring discussion |
